# Zinc-Mediated Loading and Release of His-Tagged Recombinant Proteins in Self-Assembling Peptide Coacervates

**DOI:** 10.1021/acsabm.5c02044

**Published:** 2025-12-26

**Authors:** Benjamin Clegg, Gayathri Aparnasai Reddy, Ketki Y. Velankar, Sarah M. Ostrowski, Wen Liu, Yong Fan, Ellen S. Gawalt, Wilson S. Meng

**Affiliations:** † Department of Chemistry and Biochemistry, 6613 Duquesne University, Pittsburgh, Pennsylvania 15282, United States; ‡ Graduate School of Pharmaceutical Sciences, Duquesne University, Pittsburgh, Pennsylvania 15282, United States; § Allegheny Health Network Cancer Institute, 6596 Allegheny Health Network, Pittsburgh, Pennsylvania 15212, United States; ∥ McGowan Institute for Regenerative Medicine, 536993 University of Pittsburgh, Pittsburgh, Pennsylvania 15213, United States; a Department of Pharmacy and Therapeutics, School of Pharmacy, University of Pittsburgh, Pittsburgh, Pennsylvania 15219, United States; b Center for Clinical Pharmaceutical Sciences, School of Pharmacy, University of Pittsburgh, Pittsburgh, Pennsylvania 15219, United Statess

**Keywords:** self-assembling peptide, coordination bond, long-acting injectable, bioaffinity, sustained release

## Abstract

The development of tunable systems for subcutaneous injection is currently the focus of exploratory protein formulation. For the delivery of protein biologics intended for extended release, a high fraction of the drug that escapes from the deposition site (“burst release”) may pose safety concerns. Herein, we report an injectable system of bioaffinity zinc-containing peptide coacervates in which recombinant proteins coexpressed with histidine (His)-tags can be captured and released over time. Coacervates are formed by driving self-assembling peptide (SAP), AEAEAKAKAEAEAKAKHHHHHH (EAKH6) β-sheet dimers, into cross-linking fibrils. In the presence of Zn^2+^, the fibrillization of EAKH6 is enhanced through the interaction of metal ions with histidine residues in the peptide. The Zn^2+^:EAKH6 scaffold retains His-tagged proteins both in vitro and in vivo and extends their duration of release. The results present a case study in which the Zn^2+^–[His]_6_ interaction can be used to tune the properties of supramolecular structures and the loading of His-tagged proteins.

## Graphical abstract

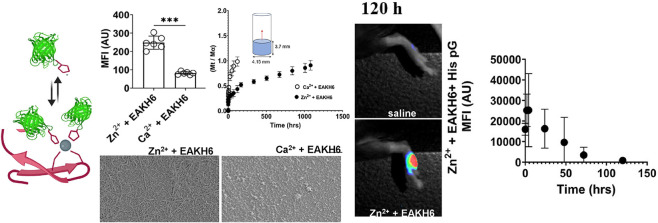

## Introduction

Recombinant proteins are considered a mainstay therapeutic platform.^1^ The rapid increase of protein-based therapeutic entities in preclinical and clinical pipelines over the past 2 decades has driven innovations in delivery strategies. One focus is the development of injectable formulations designed to extend the duration of action, thereby reducing the frequency of administration. Subcutaneous (SC) injectables have gained considerable interest due to their potential for deposition of biologically active proteins for rendering an absorption phase from the injection site into the lymphatics and subsequently into blood circulation.^2^ Although a number of injectable formulations of biologics have been approved for human use, delivery platforms that engineered with tunable drug release kinetics features remain underexplored.^3–5^

The peptide EAK16-II, AEAEAKAKAEAEAKAK (single aa code, or EAK hereafter), is a self-assembling peptide (SAP) that adopts an antiparallel β-sheet conformation and is driven into homodimers through the complementarity of alternating ionic and hydrophobic amino acid side chains. Above critical concentrations (e.g., >0.1 mg/mL) and ionic strengths (>20 mM), the β-sheet dimers stack into fibrils,^6–8^ which coalesce into cross-linking three-dimensional (3D) scaffolds.^9,10^ Depending on the specific sequences and concentrations, the variants of EAK can form similar 3D structures or phase-separated coacervates, with viscosity close to that of aqueous solutions and therefore amenable for injection using standard 25–27 gauge needles via a conventional syringe.^11–13^ Previously, we designed and characterized bioaffinity-based EAK systems for forming injectable coacervates that captured antibodies via their Fc domains.^14–17^ Although these systems are effective in extending the duration of subcutaneous (SC) deposition of IgG molecules in vivo,^16^ opportunities exist to leverage bioaffinity mechanisms for non-Fc recombinant proteins in developing injectable SC depots.^18^

Previously, we demonstrated that EAKH6, which is the EAK sequence appended with a hexa-histidine tag (H_6_) at the C-terminus, can be used as a building block for formulating His-tagged recombinant proteins into injectable coacervates.^19–22^ EAKH6 self-assembles into fibrils in physiological fluids, and the resulting scaffolds extend the duration of local depositions of SC-injected IgG molecules loaded via reversible linkers.^22^ Zheng et al. reported a system for displaying anti-CD4 IgG via anti-His-tag antibodies to anchor the complexes in a composite of EAK–EAKH6 coassemblies. Recombinant protein A/G (pAG) was used as a multivalent linker for capturing anti-CD4 IgG by binding to the Fc domain.^23^ The system is stable in vitro and extends the retention of antibodies in epithelial tumors. Wen et al. extended the applications of the EAK–EAKH6 system by localizing anti-MHC-II IgG antibodies that target donor-antigen-presenting cells in an allograft transplant site in mice. The system localizes at the graft site for up to 6 days and reduces interferon-γ (IFN-γ) levels in the tissues.^20^ The next generation of bioaffinity EAK coacervates uses a peptide derived from staphylococcal protein A (SpA). The miniaturized Fc-binding peptide Z15 consists of the first helix of the engineered SpA Z-domain fused with EAK. This bifunctional peptide, named Z15-EAK, undergoes fibrillization while exhibiting Fc-binding properties.^17^ The coacervate localizes IgG in mouse footpads for extended periods. Although these systems are effective in enhancing depot retention, their configurations also limit their applications to immunoglobulins and Fc fusion proteins.

In the current study, Zn^2+^ is used to bridge EAKH6 and His-tagged proteins (Figure 1). The affinity-based cross-linking matrix governs the loading and release of proteins driven by a theoretical binding affinity (*K*_D_) of 10^–8^ M.^24,25^ The Zn^2+^–[His]_6_ interaction is a well-characterized coordination in which one of the nitrogen atoms in histidine imidazole donates a lone pair of electrons to the Zn^2+^ orbital 4S. With each Zn^2+^ interacting with two or four histidine residues simultaneously, this dynamic interaction stabilizes the peptide β-sheet structure and enables reversible binding with His-tagged proteins (Figure 1). Herein, we present in vitro and in vivo evidence to demonstrate Zn^2+^:EAKH6 supramolecular biopolymerization as a formulation strategy for His-tagged recombinant proteins.

**Figure 1:**
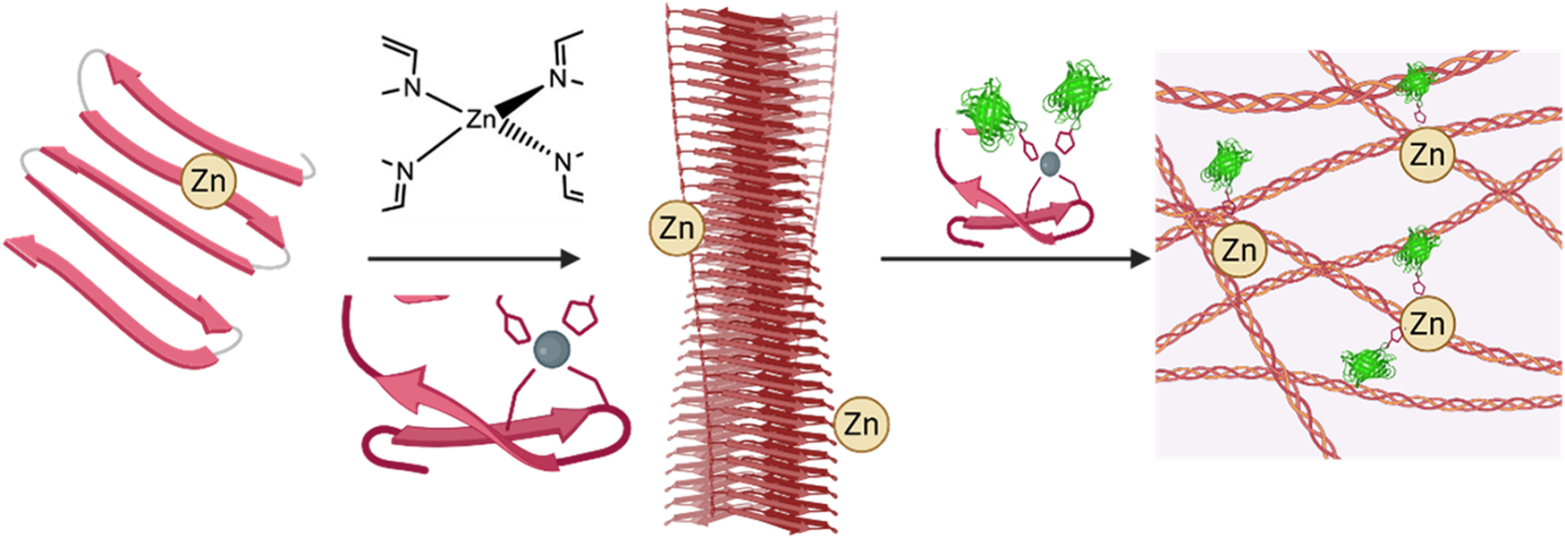
Schematic illustration of the Zn^2+^-mediated hierarchical assembly of EAKH6 peptides. Zn^2+^ coordinates with histidine residues in the peptide, promoting β-sheet formation and the alignment of peptide fibrils into an interconnected cross-linked network.

## Materials and Methods

Peptides EAKH6 (>95% purity by high-performance liquid chromatography (HPLC)) were custom-synthesized by Biomatek (Kitchener, ON, Canada) with the N-terminus acetylated and C-terminus amidated. EAK16, with a purity of >95%, was synthesized by American Peptides. Anhydrous zinc chloride, anhydrous calcium chloride, 6-methoxy-8-*p*-toluene-sulfonamidoquinoline (TSQ), and imidazole were obtained from Sigma-Aldrich. Green Fluorescent Protein coexpressed with a hexahistidine tag (His-tag) (HisGFP) was purchased from Sino Biological. Ethylene diamine tetraacetic acid (EDTA) was obtained from Fischer Scientific. Protein G coexpressed with His-tag (pG-His) was purchased from Acro Biosystems. Alexa Fluor 680 was obtained from ThermoFisher Scientific.

### Qualitative and Quantitative Analysis of EAKH6 β-Sheet Formation

The fibril morphologies of EAKH6 and Zn^2+^-bound EAKH6 at different molar ratios were assessed using a Congo red (CR) staining assay (ThermoFisher). Briefly, a 1× Congo red working solution was prepared by diluting the manufacturer-provided 10× stock in phosphate-buffered saline (PBS). Peptide samples were incubated with the dye overnight at room temperature to allow sufficient binding to β-sheet structures prior to analysis and imaged using an IX73 epi-fluorescent inverted microscope (Olympus, Centre Valley, PA). The samples were additionally measured using UV–vis spectroscopy to observe the spectral shift in the CR absorbance of Zn^2+^-bound EAKH6 from 500–540 nm. The ratio of absorbance corresponding to the bound CR:free CR was quantified and compared with the CR spectra to evaluate the effect of different Zn^2+^ to histidine ratios on EAKH6 fibril formation.

### Zn^2+^ Localization in Peptide Fibrils Using a Fluorescent Probe

Zn^2+^ retention in peptide fibrils was examined in Zn^2+^:EAKH6 and Zn^2+^:EAK at a 10:1 molar ratio of Zn^2+^ to histidine. Coacervates were prepared in a 96-well plate and incubated overnight with CR. 6-Methoxy-8-*p*-toluene-sulfonamidoquinoline (TSQ), a Zn^2+^-specific fluorescent probe, was added and equilibrated for 2 h. Fibrils were washed twice with 100 μL of deionized (DI) water to remove unbound Zn^2+^ and visualized using an IX73 epi-fluorescent inverted microscope (Olympus, Centre Valley, PA). The fibrils were further quantified for Zn^2+^ localization using the ImageJ analysis software.

### Zn^2+^ Retention in EAKH6 Fibrils

To quantify the amount of Zn^2+^ bound within the EAKH6 fibrils, samples were prepared in Eppendorf microcentrifuge polypropylene tubes with a 10:1 Zn^2+^to EAKH6 molar ratio. The coacervates were subjected to six sequential washes with DI water, followed by centrifugation to separate the supernatant containing free Zn^2+^ ions (unbound). Each wash fraction was transferred to a 96-well plate and mixed with 25.0 μL of 48.4 mM TSQ solution. Samples were then incubated for 30 min to ensure complete reaction of TSQ with free Zn^2+^ ions. Fluorescence measurements were obtained using a Tecan multiplate reader at 490 nm, corresponding to the Zn–(TSQ)_2_ complex formed from free Zn^2+^, and the extent of bound Zn^2+^ within the coacervate (pellet) was determined.

### Surface Morphologies of Fibrils and Their Interactions with Metal Ions

The surface morphologies of the fibrils formed by Zn^2+^/EAKH6 and Ca^2+^/EAKH6 (10:1 molar ratio) were examined using scanning electron microscopy (SEM). The samples were mounted on a carbon dot, rinsed gently with DI water to remove unbound metal ions, and vacuum-dried before imaging. SEM analysis was performed using a Zeiss Sigma VP SEM (Oberkochen) under varied vacuum conditions to obtain high-resolution images of the fibrillar networks. Fibril length was quantified from the SEM micrographs using the ImageJ analysis software.

Diffuse Reflectance Infrared Fourier Transform (DRIFT) spectroscopy was performed by creating 25 μL droplets of Zn^2+^:EAKH6, Ca^2+^:EAKH6, and EAKH6 alone on separate 1 cm × 1 cm stainless steel (SS316L) coupons, each in triplicate, and drying them overnight in a desiccator. The samples were rinsed with DI water and dried to remove excess water from the coupons. The samples were analyzed via a Thermo-Nicolet Nexus 470 FTIR E.S.P. spectrophotometer with a DRIFT cell attachment purged with nitrogen gas for 30 min before collecting the spectra. Spectra were obtained using 1024 scans at a resolution of 4 cm^–1^. The resulting spectra were processed using the OMNIC software.

### Protein Binding Analysis in Metal Cross-Linked Peptides

The Zn^2+^:EAKH6 coacervates were formed via coordination-driven self-assembly. The recombinant HisGFP protein (2.5 μg/mL) and 10 μL of EAKH6 (10 mg/mL) were incubated with ZnCl_2_ or CaCl_2_ at a 10:1 molar ratio of metal to histidine. Both groups were incubated for 30 min to facilitate the coordination interactions. Unbound proteins and excess metal ions were removed by rinsing the samples twice with 1 mL of DI water and centrifuging at 10,000 rpm for 5 min. The pellet (50 μL) was resuspended in an additional 50 μL of DI water to ensure uniform dispersion before analysis. The fluorescence intensity of the retained protein in the coacervate was quantified using a Tecan Infinite M1000 microplate reader (excitation 487 nm, emission 508 nm).

To evaluate the Zn^2+^-His specific coordination in Zn^2+^:EAKH6 in retaining HisGFP, three complementary experiments were performed with competitive binding of imidazole at excess concentrations, incorporating EAK (His-deficient) peptide and chelation of Zn^2+^ with EDTA.

Imidazole at high concentrations (240 mM) was added to Zn^2+^ (60 mM) to saturate the Zn^2+^-binding sites, and EAKH6 and HisGFP were added and incubated as described above. As a control, EAK (5 mg/mL) was used to prepare the coacervates as described above. In one experiment, samples were treated with EDTA (1 mM) for 30 min prior to analysis. All samples were washed and centrifuged. The amounts of proteins in the pellets were measured using a Tecan Infinite M1000 microplate reader (excitation: 487 nm; emission: 508 nm).

### In Vitro Protein Release

In vitro release kinetics of HisGFP loaded in the Zn^2+^:EAKH6 and Ca^2+^:EAKH6 coacervate synthesis were performed. Briefly, the peptide EAKH6 (10 mg/mL), HisGFP (2.5 μg/mL), and Zn^2+^ or Ca^2+^ at a 10:1 molar ratio were directly placed at the bottom of a microsert (inner diameter of 4.15 mm that holds up to 500 μL volume) with the final form being a coacervate suspension. The coacervates were incubated for 2 h to achieve equilibrium, followed by the addition of 400 μL of release media containing 0.9% NaCl and 0.1% bovine serum albumin (BSA). The microsert was then placed in an amber vial. At predetermined time points, 200 μL of release media was sampled and replaced with fresh media. The collected samples were analyzed for fluorescence intensity using a Tecan Infinite M1000 microplate reader (excitation: 487 nm; emission: 508 nm) to quantify HisGFP release over time.

### In Vivo Retention Studies

Wild-type 8–12 weeks-old female C57BL/6 mice were obtained from Hilltop Lab Animals Inc. (Scottdale, PA). Protocols for animal experimentation were reviewed and approved by Duquesne University Institutional Animal Care & Use Committee (IACUC). The mice were anesthetized with 3% isoflurane for the injection and imaging procedures. For the control group, C57BL/6 mice were subcutaneously injected with 40 μL of conjugated pG-His (10 μg/mL) in a 154 mM NaCl solution into their right footpad (FP). Alexa Fluor 680 dye conjugation with pG-His is shown in Supporting Figure S1. In the Zn^2+^/EAKH6 test cohort, 40 μL of Zn^2+^/EAKH6 (10:1 molar ratio) loaded with 10 μg/mL of pG-His was injected subcutaneously into the mice FP. Both groups were monitored for the fluorescence of conjugated pG-His and imaged using the LI-COR Pearl imaging system. The background was subtracted using the left footpad, and the fluorescence intensities were quantified. On day 3, the control group was euthanized with carbon dioxide, and the popliteal draining lymph node (DLN) at the nearest injection site was examined and imaged at 700 nm. Mice injected with Zn^2+^:EAKH6-pG-His cohort were monitored for reduced fluorescence and euthanized on day 6. Imaging was performed at 700 nm, with a resolution of 170 μm. The fluorescence intensity at the site of injection was quantified, and retention was expressed as a percentage relative to the initial signal over different time profiles.

### Computational Modeling of pG Retention at the Injection Site

Computational simulations were performed using MATLAB 2024b SimBiology to model the elimination of dissociated proteins from Zn^2+^:EAKH6 coacervates at the site of injection (FP). In vivo fluorescence intensities were quantified, and concentrations were determined using the FL intensities obtained at the time of injection, as the initial concentration injected was 10 μg/mL. The injection volume was 40 μL, which was designated as the volume of the depot compartment. A one-compartment model was built by assuming the reversible association of pG-His with the Zn^2+^:EAKH6 coacervate (Reaction 1). From the literature, histidine dissociates from Zn^2+^ with *K*_D_ at approximately 10^–8^ M; thus, the initial *k*_on_ and *k*_off_ were set as 10^6^ M^–1^·s^–1^ and 10^–2^ s^–1^, respectively. In vivo observed concentrations were fitted to a first-order decay model to estimate key pharmacokinetic parameters, including FP *K*_el_, area under the curve (AUC), and *t*_1/2_. Further, the model was simulated with different doses using the final fitted parameters and incorporating the observed and predicted data. The model was described using the following reactions and differential equations.

$$\frac{d\left(\mathrm{pG}\;{\mathrm{Zn}}^{\mathrm{II}}:\mathrm{EAKH}6\right)}{dt}=\frac{1}{\mathrm{depot}\times \left(-\mathrm{reaction}\;1\right)}$$

$$\frac{d\left(\mathrm{pG}\right)}{dt}=\frac{1}{depot\times \left(-\mathrm{reaction}\;1-\mathrm{reaction}\;2\right)}$$

$$\frac{d\left({\mathrm{Zn}}^{\mathrm{II}}:\mathrm{EAKH}6\right)}{dt}=\frac{1}{depot\times \left(\mathrm{reaction}\;1\right)}$$

Reaction 1

$$k_{off}\times pG\;Zn\left(II\right):EAKH6-k_{on}\times pG\times Zn\left(II\right):EAKH6$$

Reaction 2

$$K_{\mathrm{el}}\times \mathrm{pG}$$

### Statistical Analysis

All statistical analyses were performed using GraphPad Prism 10.0 (GraphPad, San Diego, CA), and significance was evaluated with paired and unpaired *t*-tests. Release studies and in vivo fluorescence retention were analyzed using simple linear regression. The *p-*values are indicated by asterisks, where *p < 0.05, **p < 0.01, ***p < 0.005, and ****p < 0.001, while “ns” indicates nonsignificant.

## Results

The premise of Zn^2+^:EAKH6 as a protein delivery system is that the imidazole groups in the peptide form coordination bonds with Zn^2+^. Metal–[His]_6_ affinity is routinely used in the purification of recombinant proteins coexpressed with a common tag.^26^ Herein, we present evidence demonstrating Zn^2+^-mediated loading and release of recombinant proteins using His-tagged GFP (HisGFP) and His-tagged protein G (pG-His) as model cargo proteins.

### Microscopic and Spectral Evidence of EAKH6 β-Sheet Formation

In our previous studies, thioflavin-T and circular dichroism were used to determine that EAKH6 primarily adopts a β-sheet conformation.^21,23^ In the current study, the microscopic appearance of Congo red (CR)-stained EAKH6 fibrils in the presence of Zn^2+^ was examined at varying molar ratios (Figure 2A).^27,28^ Although all samples exhibit fibrillar network characteristics of EAK-based SAPs, the Zn^2+^:EAKH6 scaffold with a 10:1 molar ratio of Zn^2+^ to histidine appears denser with a higher fluorescence intensity (Figure 2B). This qualitative observation is partially consistent with the UV–vis spectra of CR in the samples, in which the magnitude of the peak shift to longer wavelengths in the 10:1 sample was higher than that in all other samples except 20:1 (Figure 2G). Specifically, the peak shifts from the free (unbound) CR wavelength (500 nm) to the bound form (540 nm) (Table 1). The 10:1 ratio formulation was selected to move forward, given the quantitative and qualitative data.

**Table 1:** UV–Visible Absorbance of Zn^2+^:EAKH6

system	λ_1_ (nm)	λ_2_ (nm)	*A*_bound_/*A*_unbound_
Congo red	500		0.633 ± 0.003
EAKH6	500	540	0.731 ± 0.009
1:20 Zn/His	500	540	0.74 ± 0.01
1:10 Zn/His	500	540	0.74 ± 0.03
1:1 Zn/His	500	540	0.758 ± 0.007
10:1 Zn/His	500	540	0.83 ± 0.02
20:1 Zn/His	500	540	0.84 ± 0.02

**Figure 2:**
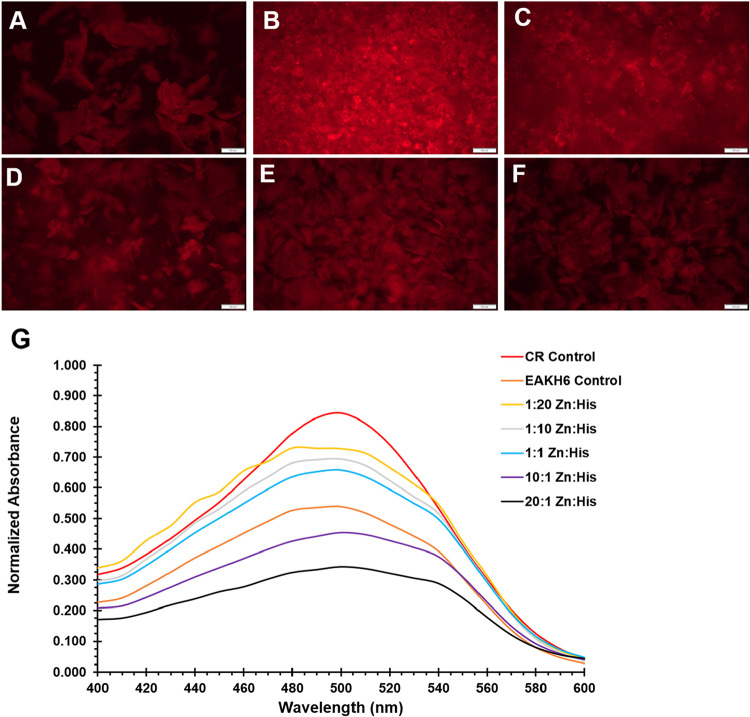
Congo red binding and fluorescence imaging of EAKH6 fibrils formed with varying Zn^2+^/EAKH6 molar ratios. (A) EAKH6 alone, (B) 10:1, (C) 20:1, (D) 1:1, (E) 1:10, and (F) 1:20 Zn^2+^/EAKH6 molar ratios. Increasing Zn^2+^ concentrations alter the fibril morphology and Congo red fluorescence. Scale bar = 100 μm. (G) UV–vis absorbance spectra of stained EAKH6 fibrils at varying Zn^2+^ concentrations.

The morphologies of the Zn^2+^-associated fibrils were examined by using SEM (Figure 3). In contrast to the EAKH6 samples mixed with Ca^2+^, the Zn^2+^:EAKH6 composite displays extensively cross-linked elongated fibrils, supporting the notion of enhanced self-assembly due to coordination (Figure 3A). The Ca^2+^:EAKH6 sample shows nonfibrillar aggregates, suggesting irregular nonspecific interactions (Figure 3B). These results support that the fibrillar structures observed in Zn^2+^:EAKH6 are mediated at least in part by specific metal–imidazole interactions.

**Figure 3:**
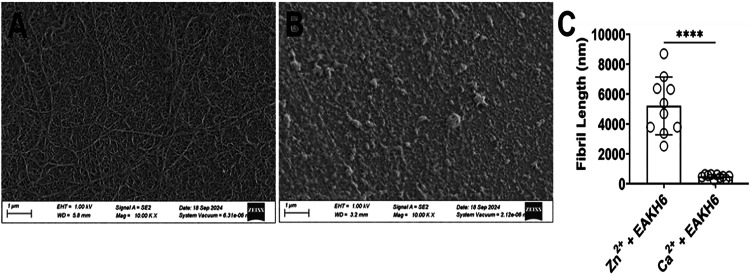
SEM micrographs of (A) Zn^2+^ + EAKH6 and (B) Ca^2+^ + EAKH6 displaying globular structures. Samples were washed with deionized water and dried. (C) Quantification of fibril lengths using ImageJ.

### Evidence of Zn^2+^ in EAKH6 Fibrils and Its Quantification

Co-localization of Zn^2+^ in the fibrils was demonstrated using TSQ, a Zn^2+^-reactive fluorogenic sensor.^29–32^ In contrast to samples prepared with Zn^2+^ and EAK, Zn^2+^:EAKH6 showed islands of TSQ fluorescence in the fibrillar structures (Figure 4A,B), suggesting [His]_6_-mediated Zn^2+^ capture. To assess the extent of Zn^2+^ retention, the samples were washed extensively with deionized water. In Zn^2+^:EAKH6, the Zn^2+^ concentration gradually decreased to 3.6% of the starting concentration after six washes, indicating the presence of bound Zn^2+^ (Figure 4D,E). Although the resolution of the imaging was not sufficient to illuminate atomic-level details, TSQ fluorescence indicates the presence of Zn in the matrix.

**Figure 4:**
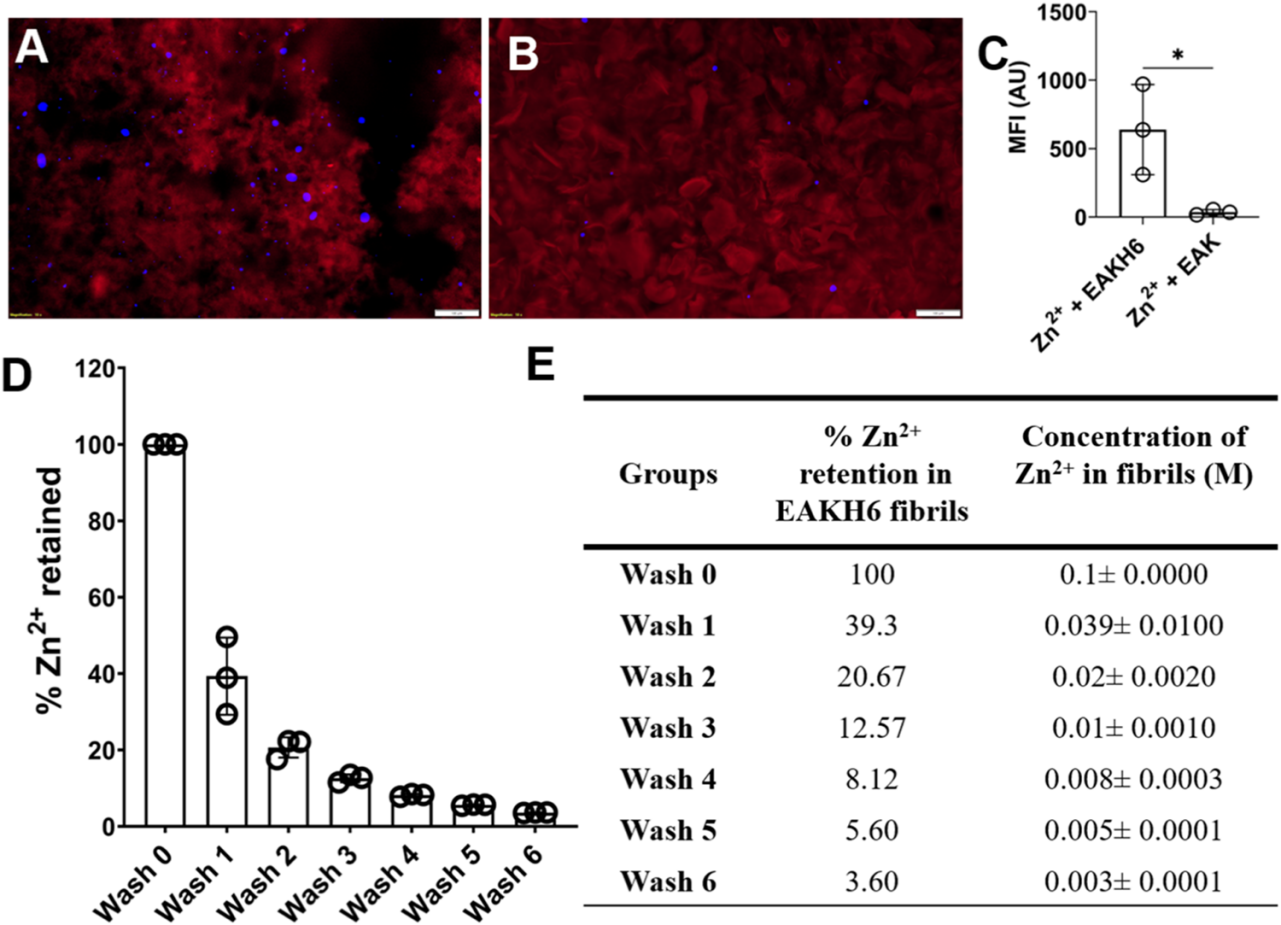
Fluorescence imaging of peptide fibrils in the presence of Zn^2+^. Illumination of samples with Congo red and TSQ in (A) Zn^2+^:EAKH6 and (B) Zn^2+^ + EAK [scale bar = 100 μm]; (C) quantification of TSQ Zn^2+^ in EAKH6 and EAK (*n* = 3), paired *t-*test; (D) quantification of Zn^2+^ EAKH6 samples in sequential washes; (E) percentage and concentration of Zn^2+^ retained.

### Spectroscopic Analysis

DRIFT was used to determine the chemical attributes of EAKH6, Zn^2+^:EAKH6, and Ca^2+^:EAKH6 (Table 2). The characteristic amide I and II bands appeared at 1620 and 1546 cm^–1^, respectively, in the spectra of all three samples^8,29,33,34^ (Figure 5A,B). Overlapping the spectra of EAKH6 and Ca^2+^:EAKH6 revealed no significant difference in the amide bands (Figure 5A), suggesting that Ca^2+^ resides in the structure via nonspecific electrostatic interactions. In contrast, the Zn^2+^:EAKH6 samples (10:1 molar ratio) show broadening of the amide II band, indicating a rearrangement of the peptide backbone (Figure 5B). A notable shift in the C–N stretching region was observed, attributed to the coordination between Zn^2+^ and the histidine residues. Additionally, the peak shift and lower intensity at 1197 cm^–1^ suggest the weakening of the N–H bond in the imidazole ring, likely due to electron redistribution upon Zn^2+^ binding.^35,36^ These results support the presence of [His]_6_-coordinated Zn^2+^ in the structures.

**Figure 5:**
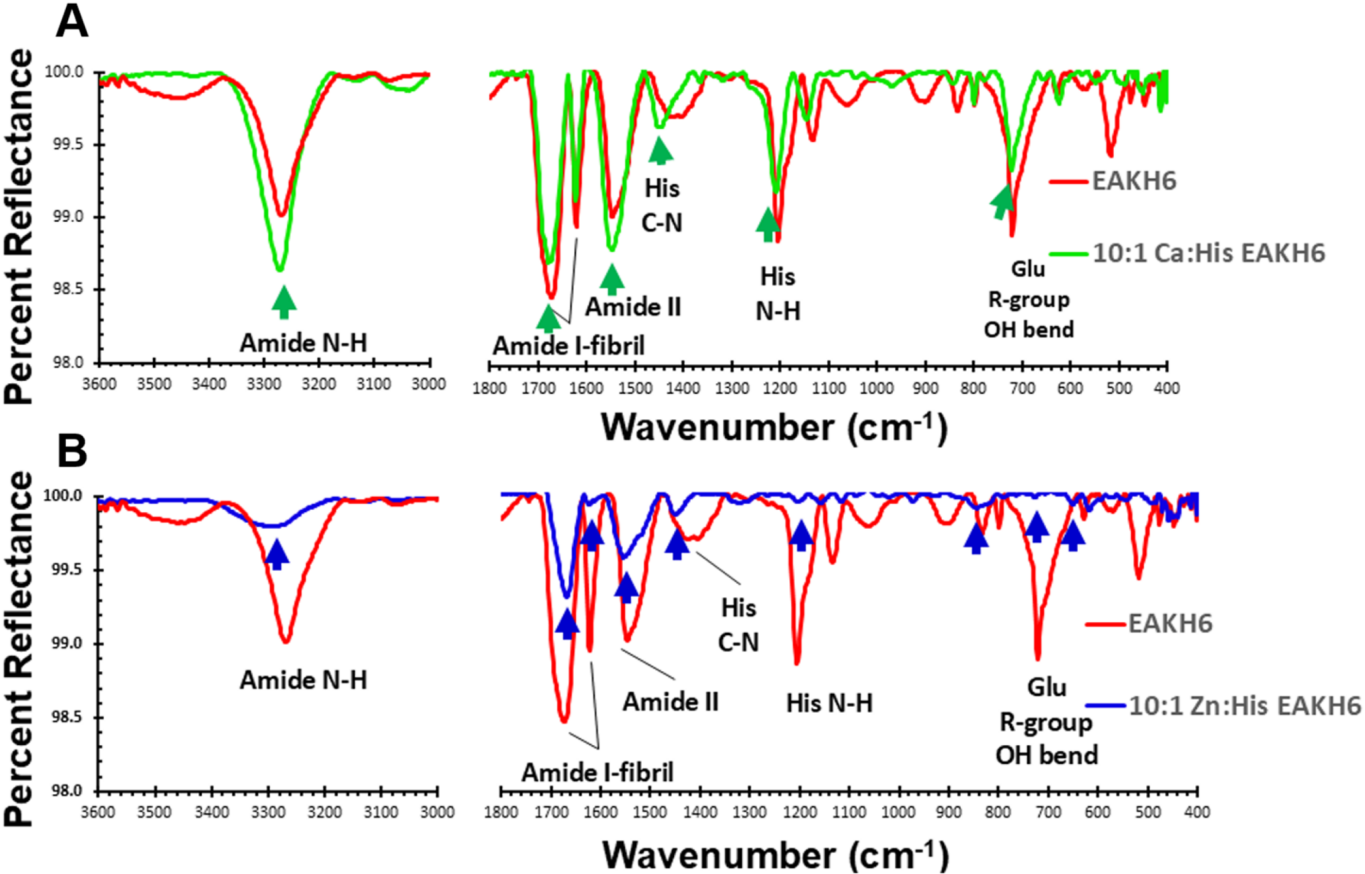
DRIFT analyses of the EAKH6, Zn^2+^:EAKH6, and Ca^2+^:EAKH6 samples. Arrows indicate amide I and II peaks at 1620 and 1546 cm^–1^, respectively; (A) comparison of EAKH6 with a 10:1 molar ratio of Ca^2+^/EAKH6, and (B) comparison of EAKH6 with a 10:1 molar ratio of Zn^2+^/EAKH6.

**Table 2:** FTIR Spectra Analysis

sample	N–H stretch	amide I (peak-1)	amide I (peak-2)	amide II	C–N	N–H	OH bend
EAKH6	3268	1670	1620	1546	1405	1203	720
intensity	1.35%	1.52%	1.06%	0.99%	0.31%	1.13%	1.11%
Zn:EAKH6	3288	1666	1621	1550	1448	1197	723
intensity	0.20%	0.69%	0.08%	0.42%	0.14%	0.06%	0.02%
Ca:EAKH6	3270	1673	1623	1546	1444	1207	721
intensity	0.98%	1.28%	0.87%	1.21%	0.37%	0.80%	0.67%

### Zn^2+^-Dependent Loading of HisGFP in EAKH6 Coacervates

The capacity of Zn^2+^:EAKH6 to capture His-tagged proteins was evaluated by using HisGFP (Figure 6A–C). Two molar ratios (10:1 and 1:1) of metal to EAKH6 were examined using a pulldown approach based on centrifugation. Based on the fluorescence intensities measured in the pellets, the Zn^2+^/EAKH6 (10:1) samples show significantly higher protein binding, approximately 74.5% ± 3.82 after washing, in contrast to 5.7% ± 1.35 in Ca^2+^:EAKH6, which was included as a negative control since calcium does not coordinate with histidine residues. The capture was reduced to 16.6% ± 2.57 in 1:1 Zn^2+^/EAKH6 and 4.8% ± 0.5 in the Ca^2+^-mixed EAKH6 samples.

**Figure 6:**
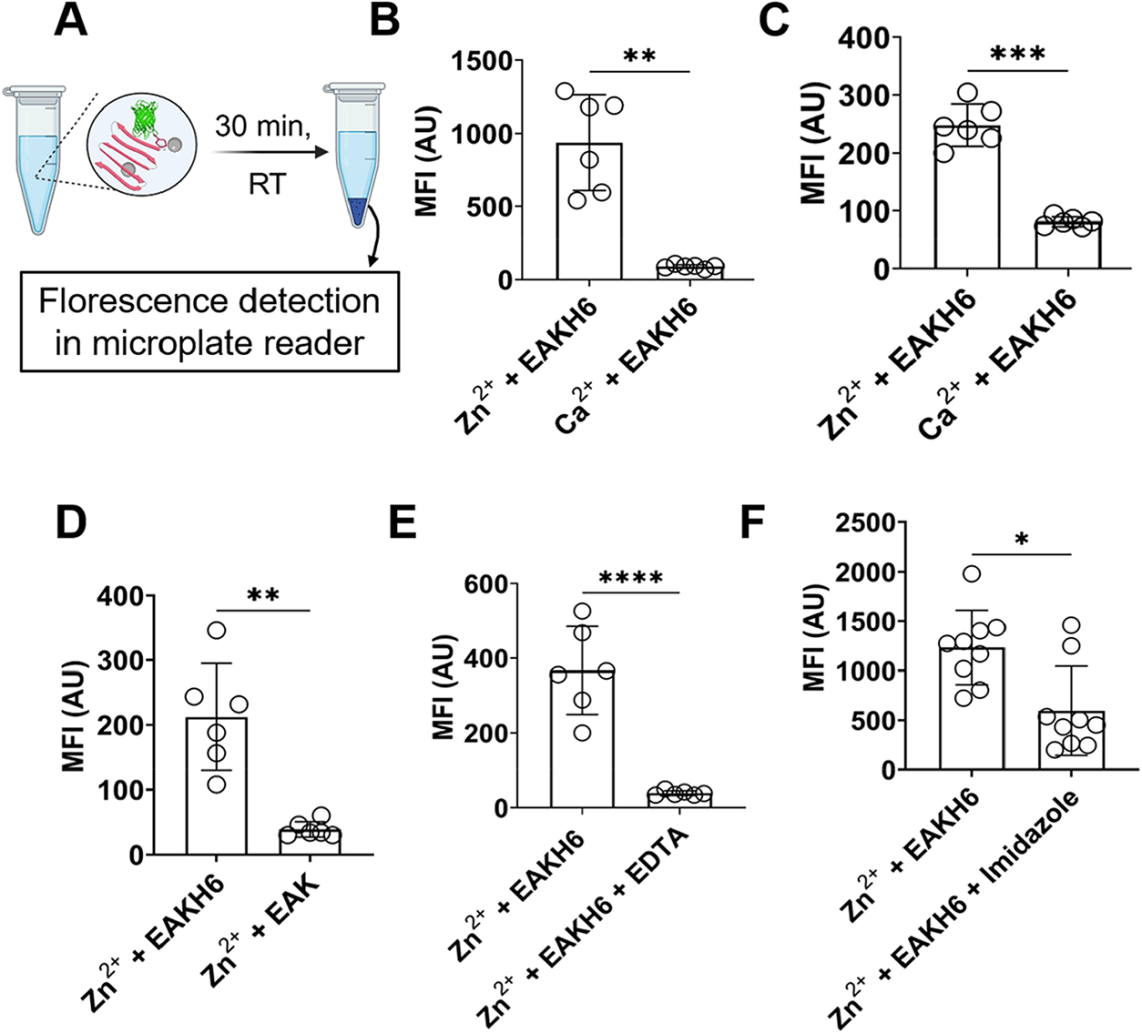
Loading capacity and specificity of His-tagged green fluorescent protein (GFP) in Zn^2+^:EAKH6 coacervates and controls. (A) Pulldown assay based on the centrifugation of GFP and metal fibrils. Fluorescence measured in fibril composites of Zn^2+^ or Ca^2+^ at metal-to-peptide molar ratios of (B) 10:1 and (C) 1:1, and controls, including (D) EAK, (E) with EDTA, and (F) with excess imidazole (240 mM). Unpaired *t*-test: ****p < 0.0001, ***p < 0.0001, **p < 0.001, and *p < 0.01. Schematic (A) was created in BioRender via an academic license.

These results suggest that the loading of His-tagged GFP is mediated by Zn^2+^ in the composite. Three additional lines of evidence support the notion of Zn^2+^–[His]_6_-specific loading (Figure 6C–E). A 52 ± 6.04% reduction in HisGFP loading was observed when an excess concentration of imidazole (240 mM) was present in the Zn^2+^:EAKH6 samples, presumably due to competition of the species for Zn^2+^.^37,38^ Using EAK instead of EAKH6 resulted in an 80% ± 0.9 decline in the loading of HisGFP. Lastly, adding EDTA to the mixture, which chelates divalent metals,^39,40^ led to an 88 ± 0.52% reduction in loading. Taken together, these results indicate that His-tagged proteins can be loaded into the fibrillar coacervates via coordination interactions among EAKH6, Zn^2+^, and HisGFP (Figure 1).

### Release Kinetics of HisGFP from Zn^2+^:EAKH6

The in vitro release profiles of HisGFP from Zn^2+^:EAKH6 and Ca^2+^:EAKH6 were monitored for 45 days. The release of the protein from Zn^2+^:EAKH6 coacervates followed a biphasic pattern in which 35% ± 0.06 of the loading concentration was released in the first 3 days, followed by a second slower phase in which 55% ± 0.09 was released from day 3 to day 45 (*n* = 3). In contrast, almost all of the HisGFP was loaded (98% ± 0.09) in the Ca^2+^:EAKH6 samples and released in 5 days (Figure 7A,B). These results are consistent with the reversible binding of Zn^2+^-coordinated His-tagged proteins, as evidenced by the delayed release of HisGFP and the apparent diffusion coefficient (Figure 7C).

**Figure 7:**
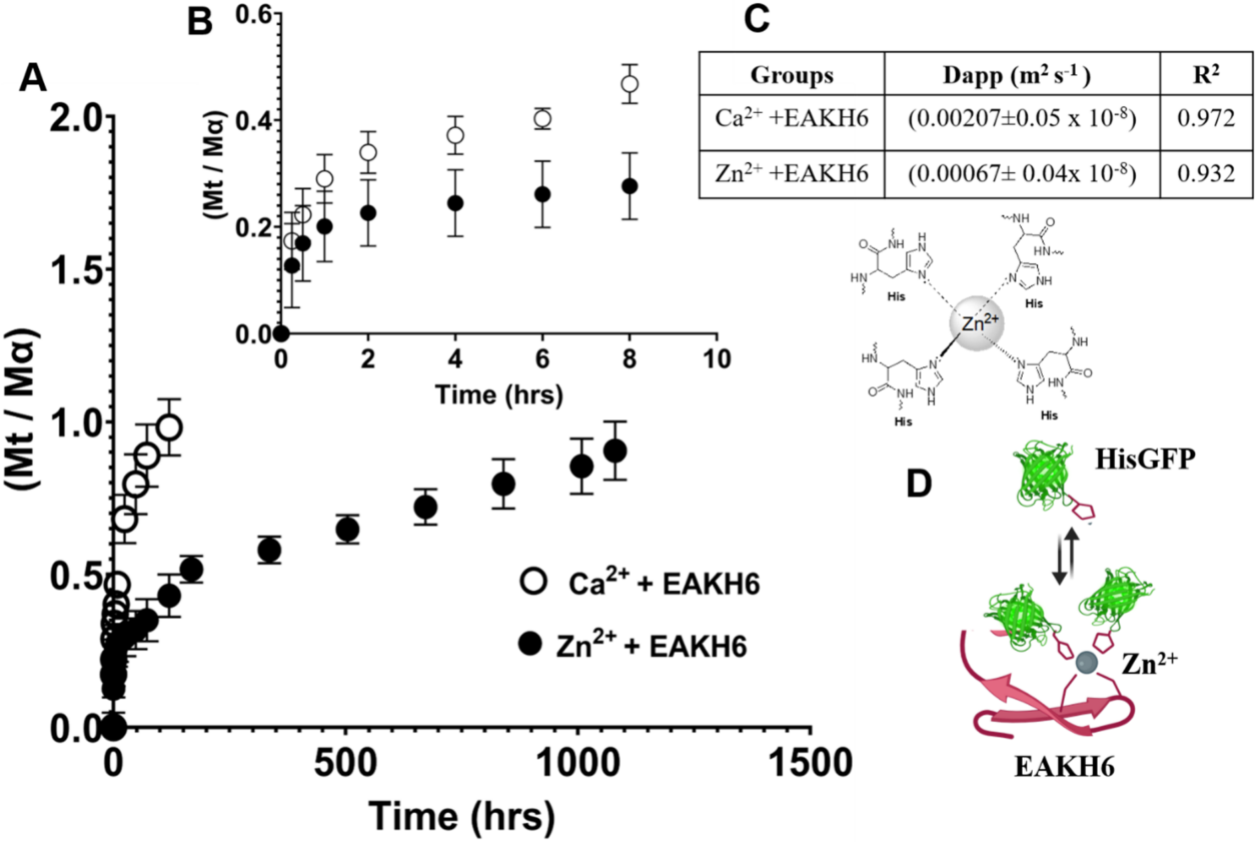
Release kinetics of His-tagged GFP from Zn^2+^ or Ca^2+^-containing peptide coacervates. (A) Cumulative release of HisGFP performed in triplicate (*n* = 3) for 45 days. (B) Initial release pattern of HisGFP up to 8 h used to determine apparent diffusivity. (C) Fick’s second law of diffusion was used to obtain the apparent diffusion (*D*_app_), where $\left(\frac{\mathrm{Mt}}{M\propto }\right)$ is the fractional release of HisGFP a*t* time *t*. Zn^2+^:EAKH6 exhibited a lower rate of diffusion of 0.00067 than Ca^2+^:EAKH6 of 0.00207 (10^–8^ m^2^ s^–1^), respectively. The release is assumed to be one-dimensional diffusion, and that the fibrillar scaffold neither swells nor degrades. (D) Schematic illustration of unbound protein release from coacervates (created in BioRender via an academic license).

### In Vivo Retention of His-Tagged Protein G in Zn^2+^:EAKH6

The capacity of Zn^2+^:EAKH6 to deposit with globular proteins in vivo was examined by injecting a near-infrared dye-conjugated His-tagged protein G (pG-His) into the SC space of the footpad in wild-type C57BL/6 mice. His-tagged protein G, or “pG”, has a theoretical *M*_w_ = 26.8 kDa, and isoelectric point (pI ≈ 5.5–6.0) close to those of His-tagged GFP (MW ≈ 27.8 kDa, pI ≈ 5.5–5.8), thus providing a reasonable extrapolation between the in vitro and in vivo studies. Both proteins are models of small globular protein biologics that have been developed into biotherapeutics and are very close to those of similar molecular weight and isoelectric point to His-tagged GFP (MW ≈ 27.8 kDa, pI ≈ 5.5–5.8), thus providing a reasonable extrapolation between the in vitro and in vivo studies. Both proteins were used as models for small globular protein biologics.^41^ The footpad was selected because it is a well-defined anatomical site for studying local protein deposition and lymphatic draining.^42^ The mouse footpad consistently drains into a single lymph node (popliteal lymph node); therefore, it is conducive for tracking analysis. At the experimental endpoint, the popliteal lymph node was exposed and imaged (Figure S3).

A cohort (*n* = 3) of mice that received pG-His in saline was used as the control groups. Live imaging was performed at the indicated time points after a single injection (Figure 8A). After 24 h, only about 16% of the initial mean fluorescence intensity (MFI) remained at the injection site in the control group, reaching background fluorescence at 72 h. In contrast, approximately 64% of the initial MFI was measured at the injection site after 24 h in mice that received pG-His formulated in Zn^2+^:EAKH6, with the fluorescence remaining above the background for up to 120 h (Figure 8B–D). These results demonstrate that Zn^2+^:EAKH6 provides a loading mechanism for the in vivo deposition of His-tagged proteins. The retention of pG-His coincides with delayed absorption into the draining popliteal lymph nodes (Figure S3).

**Figure 8:**
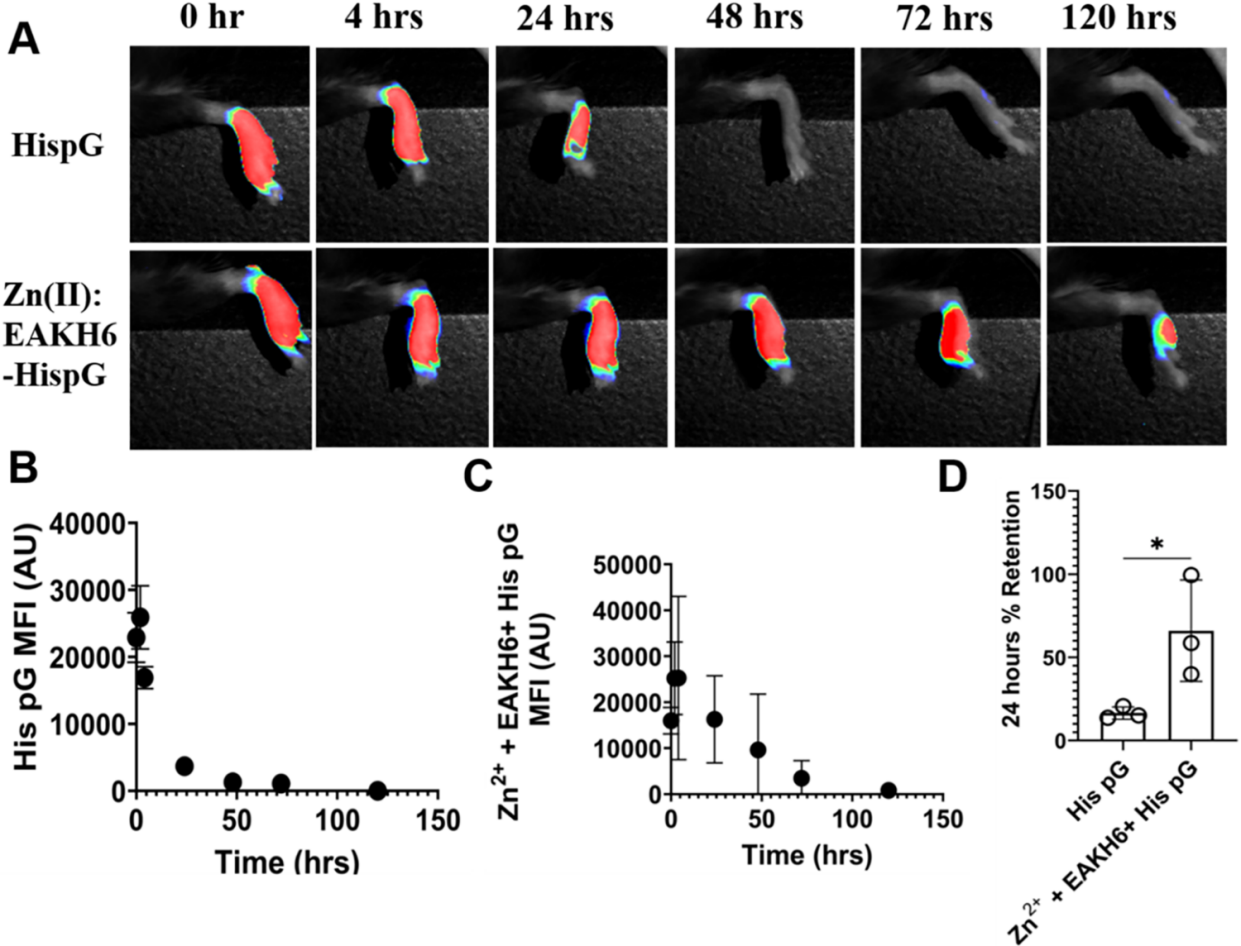
In vivo retention of pG-His injected subcutaneously into the mouse footpad. (A) Fluorescent images of the footpad from 0 to 120 h obtained at 700 nm, with a resolution of 170 μm. (B, C) Quantification of fluorescent intensities in mice injected with dye-conjugated pG-His formulated in (B) saline or (C) Zn^2+^:EAKH6. (D) Retention of pG-His (*n* = 3). Unpaired *t*-test with *p < 0.01.

### Computational Modeling of pG-His Elimination Kinetics at the Injection Depot

The in vivo data was fitted to a kinetic model function based on the reversible binding of pG-His from Zn^2+^:EAKH6 (Figure 9), which was assumed to remain in the depot, and only the dissociated pG-His left the compartment (Figure 9A). The observed fluorescence intensities were converted to concentrations by using initial fluorescence versus time profiles.^14^ The overlay of the experimentally observed data and predicted levels is closely aligned. The predicted values were used to calculate *K*_el_, and the trapezoidal method was used to obtain the total exposure parameter Area Under the Curve (AUC_0–∞_).^43^ The exposure of pG-His was computed to be 184 (μg·h)/mL for the control group and 410 (μg·h)/mL for the Zn^2+^:EAKH6 group, respectively; the estimated parameters included *K*_el_, AUC, and *t*_1/2_ (Table 3). Using the one-compartmental model, the impact of different coacervate doses on the retention of proteins at the injection site was simulated. As the dose increased (0–100 μg/mL), the calculated AUC at the injection site increased in a linear fashion (Figure 9C). In our system, protein loading is a function of the matrix density of EAKH6 fibrils and, secondarily, the molar concentration of bound Zn^2+^ coordinating up to four histidine residues.

**Figure 9:**
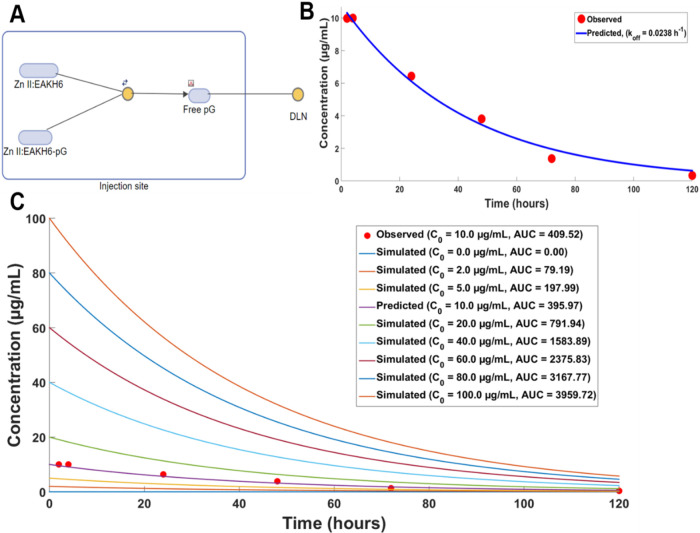
Modeling of pG-His elimination from the footpad depot. (A) One-compartmental model governed by first-order reaction kinetics and reversible binding between the cargo protein and metal–peptide coacervate; (B) fitting of imaging data to first-order elimination; (C) simulation of loading concentrations of the cargo protein (0–100 μg/mL) and prediction of AUC_0–∞_. Schematics were generated using MATLAB 2024.

**Table 3:** Computed Pharmacokinetic Parameters

groups	*K*_el_ (h^–1^)	AUC_0–∞_ (h)	*t*_1/2_ (h)
HispG	0.0712	184	9.73
Zn(II):EAKH6 + HispG	0.0238	409.52	29.12

The differentiating feature is that the loading capacity (per mg) of the protein in biomaterials (Zn^2+^:EAKH6) is conducive to scale-up. At the low protein concentration used in vivo (10 μg/mL), the available Zn^2+^ sites are sufficient to capture most proteins. At higher protein concentrations, such as 100 mg/mL, the 10:1 Zn^2+^/His molar ratio would be saturated, reducing the relative protein loading. To accommodate higher concentrations, both EAKH6 fibrils and Zn^2+^ would need to be proportionally increased.

## Discussion

Herein, we present in vitro and in vivo evidence to support leveraging Zn^2+^–[His]_6_ coordination as a mechanism to extend the release of proteins from a matrix of peptide fibrils. The reversible binding of Zn^2+^ and His-tags has been utilized extensively for protein purification.^44–48^ Indeed, most recombinant proteins are coexpressed with a His-tag, making the design applicable to diverse sets of biotherapeutics. The effects of Zn^2+^ were observed at two levels: First, the metal enhances the phase separation of EAKH6 into β-fibril coacervates (Figure 2A–F). Second, the observed reversible protein loading and release are mediated through the interaction between Zn^2+^ and [His]_6_ in EAKH6 and model cargo proteins (Figure 6A,B).

Our study focused on demonstrating the specificity of the Zn^2+^ and [His]_6_ interaction because, although metal-His-tag-based supramolecular polymerization is employed in biomaterial design, the specific role of metal–organic coordination remains key mechanistic evidence. We present data showing Zn^2+^ [His]_6_-specific protein capture: composites consisting of excess imidazole, the addition of EDTA, substitution of EAKH6 with EAK, and replacement of zinc with calcium all resulted in reduced loading. The contrast between Zn^2+^ and Ca^2+^ is particularly informative. Zn^2+^ has an empty valency shell at the 4s orbital and fully filled d-orbitals, which accept a lone pair of electrons from nitrogen in the histidine side chain to form a coordination bond, adopting a tetrahedral geometry.^49–51^ Conversely, Ca^2+^ lacks a d-orbital and a larger atomic radius, does not coordinate with [His]_6_, but could form ionic interactions with the carboxylate of the glutamic acids in EAKH6. The nonspecific interactions may explain the lack of elongation of EAKH6 fibrils and the poor loading of His-tagged proteins in Ca^2+^:EAKH6.

The extended release of HisGFP from Zn^2+^:EAKH6 coacervates is attributed to the coordination bond that creates a dynamic network of reversible binding of Zn^2+^ to the histidine residues, which allows the diffusion of the protein.^4,48^ Zn^2+^:EAKH6 and HisGFP undergo association and dissociation constants that govern the transient retention and diffusion of HisGFP from Zn^2+^:EAKH6.^52^ The rapid release of the cargo protein from Ca^2+^:EAKH6 is likely due to nonspecific electrostatic interactions, as the rate of diffusion is governed by the network density and solute concentration gradient. Optimizing the metal-to-peptide ratio with affinity-based interactions allows an increased cross-linking network of fibrils, which sustains protein diffusion from the coacervate. Fluorescence imaging and TSQ quantification reveal the persistent association of Zn^2+^ with EAKH6. The Zn^2+^ ions may stabilize the EAKH6 β-sheet structures, thereby reducing the intrinsic disorder of the tag and facilitating the integration into the β-fibrils.

DRIFT analysis further supports the interaction between Zn^2+^ and EAKH6 via the imidazole of the hexahistidine region. This is based on the peak shifts and reduced intensity in N–H stretching and C–N stretching at 3288 and 1448 cm^–1^, respectively, compared to EAKH6 with Ca^2+^.^53,54^ The altered electronic density of the imidazole nitrogen in histidine could arise from the coordination with Zn^2+^. The Ca^2+^:EAKH6 group displayed peaks and intensities similar to those of EAKH6, indicating electrostatic interactions without structural changes in the peptide.

In vivo studies confirmed the Zn^2+^:EAKH6 retention of pG-His for 5 days at the injection site and controlled rapid diffusion. Meanwhile, pG-His in saline diffuses rapidly within a day of administration. The prolonged duration of the fluorescence intensity observed in Zn^2+^:EAKH6 highlights the advantage of affinity-based systems.^55^ In our in vivo experiments, SC-injected Zn:EAKH6, loaded with His pG, shows no visible signs of local inflammation, and irritation and swelling were noticed at the injection site for Zn2+:EAKH6 loaded with pG. The mice exhibited normal activities. This is consistent with our previous studies that show that injecting EAKH6 into the SC space in mice did not elicit acute toxicities.^7,22^ Self-assembling peptides have been reported to be nonimmunogenic. The injection volume (40 μL) and protein concentration (10 μg/mL) are within the range of commonly reported SC delivery platforms. Zn^2+^ is an essential trace element in the body and is biocompatible. Our preliminary studies show no local adverse effects, and toxicology studies are required for additional assessment of the safety profile.

## Conclusions

Tunable Zn^2+^:EAKH6 soft materials can be used to capture and release His-tagged recombinant proteins. The results presented provide a mechanism for developing new formulations of long-acting injectable therapeutic proteins.

## Supplementary Material



## Notes

Dye conjugation of recombinant protein G; enzyme linked immunosorbent assay (ELISA)-based quantification of Alexa Fluor 680-labeled protein G; purification and characterization of conjugated pG; confirmation of conjugated protein G and His-tag binding with IgG; UV–visible spectra analysis of conjugated pG-His with Alexa Fluor 680 and its imaging (Figure S1); determination of apparent diffusion using approximation of initial release of HisGFP in vitro (Figure S2); images of popliteal lymph nodes in wild-type C57BL/6 mice (Figure S3) (PDF)

## #

Amgen Inc., 1 Amgen Center Dr, Thousand Oaks, California 91320, United States
