# Supplementary material for: Zinc-Mediated Loading and Release of His-Tagged Recombinant Proteins in Self-Assembling Peptide Coacervates
Source: ACS Appl Bio Mater. 2025 Dec 26;9(2):1064–75. doi: 10.1021/acsabm.5c02044 (PMC12820974; doi:10.1021/acsabm.5c02044)
Supplement: Supplementary file 1 [file mt5c02044_si_001.pdf]

Zinc-Mediated Loading and Release of His-tagged Recombinant Proteins in Self-Assembling Peptide Coacervates

Authors

Benjamin Clegg[1,#], Gayathri Aparnasai Reddy[2,#], Ketki Y. Velankar[2,&], Sarah M. Ostrowski[4], Wen Liu[3], Yong Fan[3], Ellen S. Gawalt[1,4,*], and Wilson S. Meng[2,4,*]

Affiliations

[1]Department of Chemistry and Biochemistry, Duquesne University, PA 15282, USA
[2]Graduate School of Pharmaceutical Sciences, Duquesne University, Pittsburgh, PA 15282, USA
[3]Allegheny Health Network Cancer Institute, Allegheny Health Network, Pittsburgh, PA, USA
[4]McGowan Institute for Regenerative Medicine, University of Pittsburgh, PA 15213, USA

[#]These two authors share co-first authorship
*Corresponding authors: meng@duq.edu, gawalte@duq.edu
[&]Current address: Amgen Inc., 1 Amgen Center Dr, Thousand Oaks, CA 91320

Supporting Information

Dye conjugation of recombinant Protein G

Recombinant protein G, His tag (pG-His) was covalently conjugated with Alexa flour 680 NHS ester at a final dye-to-protein ratio of 4:1 mol/mol. The reaction was carried out in an alkaline pH of 9.5 and was incubated for 2 hrs in a dark place. Conjugated Protein was purified with a Molecular Probes Antibody Conjugate purification kit, employing a gravity-fed size exclusion column with Biogel P-30 resin, which is suitable for purifying pG-His (30 kDa). The concentration of conjugated Protein and degree of labelling was calculated as

$$\text{Protein conc (M)} = \frac{(\text{A280} - \text{A680} * 0.05)\text{dilution factor}}{\text{Molar extinctionn coefficient of dye} * \text{pG conc (M)}}$$

$$\text{Degree of labeling} = \frac{\text{A280} * \text{dilution factor}}{\text{Molar extinctionn coefficient of dye} * \text{pG conc (M)}}$$

ELISA-based quantification of AlexaFluor 680 labelled protein G

Alexa flour 680 labelled recombinant pG-His (conjugated pG-His) was quantified with ELISA by immobilizing the human IgG to a Li-Cor black 96-well plate. Conjugated pG-His and control groups were added to the coated surface and incubated for 30 minutes, refer to Figure X. Unbound conjugated pG-His was rinsed, and the fluorescence intensity was analyzed with Tecan M1000 Infinite (excitation: 680 nm, emission: 702 nm). Additionally, the 96-well plate was imaged with the Odyssey imaging system at 700 nm with 170 micron resolution to access the fluorescence signals in all groups tested. The results were analyzed to determine the extent of conjugated pG-His binding to human IgG.

Purification and characterization of conjugated pG

The conjugation of pG-His with Alexa flour 680 by NHS ester was confirmed with UV-Visible spectroscopy by measuring the absorbance at 280 nm and 680 nm. Absence of 280 nm peak indicates the unconjugated fraction of dye to protein. Additionally, fluorescence analysis of conjugated pG-His was performed using odyssey fluorescence imaging microscopy at 700 nm, confirming the fluorescent signal of the conjugated dye. The degree of labelling determined, as mentioned above and was found to be 4:1 molar ratioe of dye to protein. The concentration of conjugated pG-His was found to be 0.166mg/mL. These results confirm the presence of pG-His and functionality of dye was preserved.

Confirmation of conjugated protein G, His-tag binding with IgG

The conjugation of pG-His with Alexa Flour 680 was further validated by assessing its binding to human IgG using ELISA. The surface of the wells was coated with IgG and was introduced with different groups. Fluorescence intensity measurements with Tecan M1000 infinite exhibited significantly higher fluorescence in conjugated pG-His group with IgG, compared to controls including Alexa flour 680 with IgG, pG AF alone, IgG with SA IRDye 680. These results confirm the binding of conjugated pG-His with immobilized human IgG. Moreover, odyssey imaging further validated the fluorescence signal and displays higher fluorescence in wells with conjugated pG-His with IgG. Background fluorescence was observed with conjugated pG-His alone and other control groups indicate the effective washing and binding specificity with conjugated pG-His to human IgG. The fluorescence differences among experimental groups explain the conjugation of pG- His with Alexa Flour and binding specificity with human IgG.

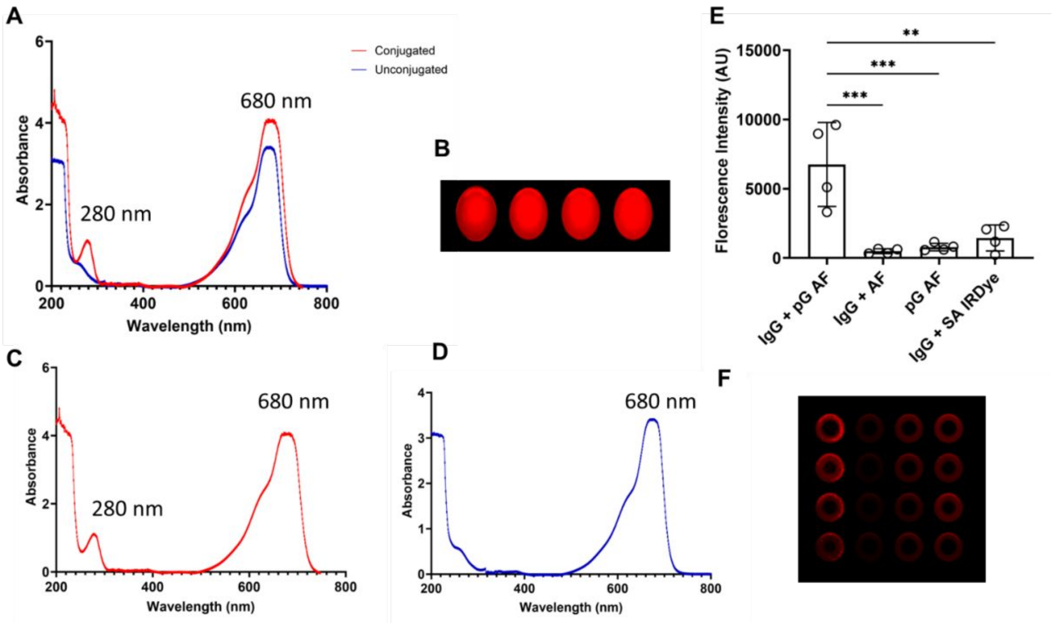

Fig. S1 UV-visible spectra analysis of conjugated pG-His with Alexa flour 680 and its imaging. A) Overlay of absorbance of free dye and pG-His conjugated with the dye, B) Fluorescence of conjugated dye with pG-His, UV absorbance of C) dye-conjugated pG-His and D) free dye at 280nm and 680 nm, and E) functional assay of dye-conjugated pG-His showing its binding to IgG and F) images of the samples.

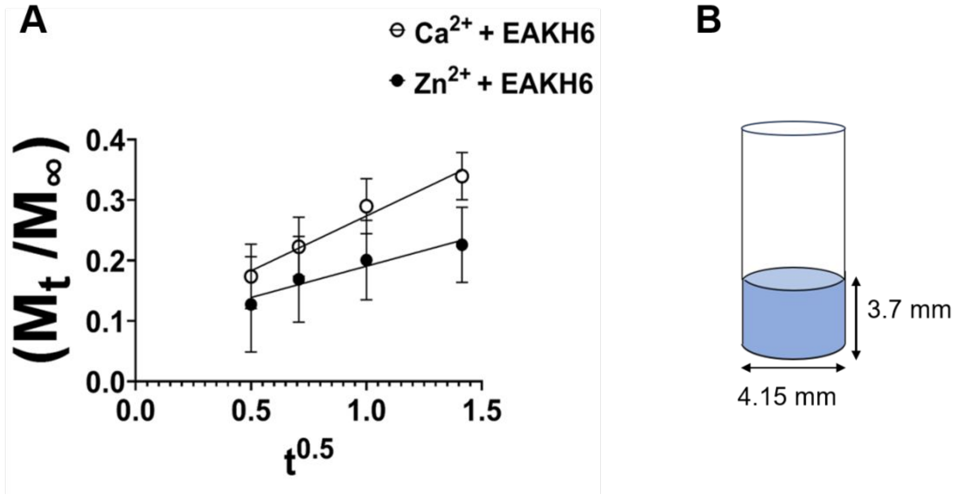

Fig. S2 Determination of apparent diffusion using approximation of initial release of HisGFP *in vitro*. A) The Zn^{2+} /Ca^{2+}: EAKH6 group apparent diffusion. B) The height of the formulation in vial 3.7mm with inner diameter 4.15 mm. Fluorescence at each time point was determined by measuring the aliquot medium using a microplate reader. Data represent samples in triplicate.

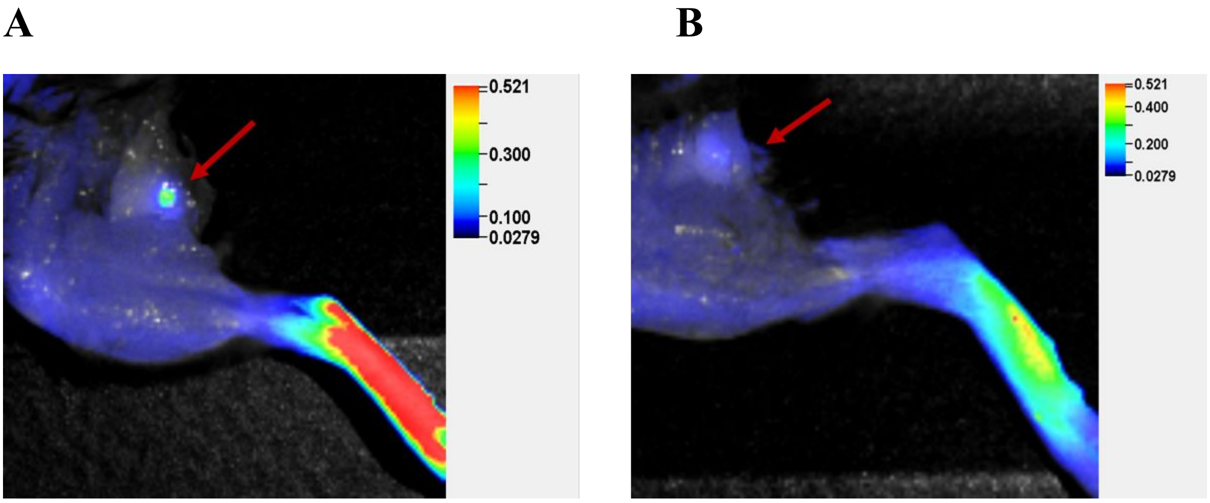

Fig. S3 Images of popliteal lymph nodes in wild-type C57BL/6 mice at A) 144h for Zn^{2+}:EAKH6 group and B) 48h saline group post injection. The tissue was exposed by excising the skin before the image capture (at 700 nm channel in 170-micron resolution).
